# Diagnostic accuracy of cone-beam CT compared with panoramic images in predicting retromolar canal during extraction of impacted mandibular third molars

**DOI:** 10.4317/medoral.19930

**Published:** 2014-12-05

**Authors:** Yıldıray Sisman, Ahmet Ercan-Sekerci, Mehtap Payveren-Arıkan, Halil Sahman

**Affiliations:** 1 Associate Professor and Chair, Department of Oral and Maxillofacial Radiology, Faculty of Dentistry, Erciyes University, Kayseri, Turkey; 2 Assistant Professor, Department of Oral and Maxillofacial Radiology, Faculty of Dentistry, Erciyes University, Kayseri, Turkey; 3 Physicist, Department of Dentomaxillofacial Radiology, Faculty of Dentistry, Erciyes University, Kayseri, Turkey; 4 Assistant Professor, Department of Oral and Maxillofacial Radiology, Faculty of Dentistry, Abant İzzet Baysal University, Bolu, Turkey

## Abstract

Objectives: The clinical significance of the existence of a retromolar canal and of its neurovascular content is not yet clear.The aim of the present study was to assess the visibility, diameter and course of the mandibular retromolarcanal (MRC) using cone beam computed tomography (CBCT) scan -had been taken for pre-operative radiographic evaluation of impacted mandibular third molars- compared to panoramic radiographs. Study Design: Subjects eligible for study enrollment were those who underwent preoperative CBCT scan for the extraction of impactedmandibular third molars were determined to be extremely close to the mandibular canal on panoramic radiographs. Radiographs were screened for the presence and course of retromolar canals, and linear measurements. Results: 947hemimandibles in 632 patients were examined.A total of 253 MRCs (144 left, 109 right) were detected with CBCT images (26.7%). Only 29 of these canals were also seen on the corresponding panoramic radiographs. Most MRCs had a vertical course (type VI, 28.46%), followed by slightly curved (type I, 26.09%). The visibility of the MRC on the OPGs, according to the increase in the diameter, was not statistically significant for both sides (*p*>.05).Statistically difference were found for the width at the point of origin from the mandibular canal (*p*: .037), the mean distance from the MRC to the second molar (*p*: .042) and height of MRC when compared the gender. Conclusions: The findings suggest that the MRC isn’t a rare anatomical structure. This study therefore clearly establishes the incidence and importance of the MRC. The detection of the presence of the MRC using CBCT may be crucial for extraction of mandibular third molars.

** Key words:**Accessory innervation, cone beam computed tomography, mandibular anatomy, panoramic radiographs, retromolar canal, retromolar foramen.

## Introduction

The mandibular retromolar canal (MRC) is a rare anatomic variation found in the retromolar triangle, a small triangular-shaped region posterior to the third molar tooth in the mandible (1). Identifying the position and configuration of mandibular canal variations are important in surgical procedures including sagittal split osteotomy, removal of impacted third molars, and implant placement (2). The neurovascular content of the MRC is very important for surgical procedures involving the retromolar area and there has been a lack of information on this subject (3).

The clinical significance of the existence of a MRC and of its neurovascular content is not yet clear. There have been limited articles about both the anatomy and the description of MRC in anatomy or surgery textbooks until recent research in this area. Knowledge of this anatomical variation may prevent complications in the anesthesia and surgical procedures in this area and serve as an anatomical landmark for ethnic identification (4).

Orthopantomographs (OPGs) provide primary and significant data on the oral and maxillofacial region (5). Nevertheless, OPGs and other two-dimensional radiographs fail to show the buccolingual aspect and cross-sectional slices which are important for presurgical assessments (6). Obtaining reliable measurements is also not possible with OPGs because of other disadvantages such as patient positioning, magnification distortion and superposition of the anatomical structures (7). Hence, the detection of the MRCs and other anatomical variations (mandibular incisive canal, accessory mental foramen, accessory mandibular canal, etc.) using OPGs may provide limited and misleading data (5,8).

Cone beam computed tomography (CBCT) technology is a substantial impact on maxillofacial imaging that has been used in several areas of dentistry because it shows three-dimensional (3D) images of dental structures in addition to providing clear structural images with high accuracy (9). It has enabled us better to visualize the anatomy of the mandibular canal and ramified canals (2). The aims of the present study were to assess the enhanced visibility of the MRC in CBCT images compared with OPGs and to assess the visibility, diameter and course of the MRC using CBCT.

## Material and Methods

The materials for this study were obtained from the Department of Oral and Maxillofacial Radiology, Faculty of Dentistry, X University. Patients were referred for CBCT scan (NewTom 5G, QR, Verona, Italy) following a preliminary diagnosis of relationship between the mandibular canal and impacted mandibular third molars by the referring practitioner from a panoramic radiograph (Instrumentarium OP200D digital, Tuusula, Finland; 66-85 kVp, 10-16 mA, 14.1 exposure time).

This retrospective study sample consisted of 632 individuals (947 mandibular third molars) that showed the retromolar area of the mandible. Two experienced dentomaxillofacial radiologists evaluated the corresponding panoramic and CBCT images to examine for the presence or absence of the mandibular retromolar canal and foramen. When disagreement existed between the two observers, consensus was reached by discussion. The CBCT images were processed with NNT software (Version 3.0, Verona, Italy). The software is totally designed by New Tom. The NNT advantage: -NNT allows the user to select from among various “application modes” based on their specific professional needs. -New Tom images are compatible with most major third-party software programs on the market. -The new module dedicated to implant preparation allows the specialist to accurately determine the most suitable type of implant to be inserted, as well as its precise positioning. -The software features a vast library of implant models in order to facilitate the simulation of the surgical procedure. It is even possible to create custom implant mode.

Axial, sagittal, cross-sectional and panoramic images were reconstructed for all semi-mandibles, and 3D reconstruct ions were used as necessary. The panoramic radiographs were only analyzed on the side (left, right, or both sides) for which a CBCT scan had also been performed according to the indication of patient referral.

**Figure 1:**
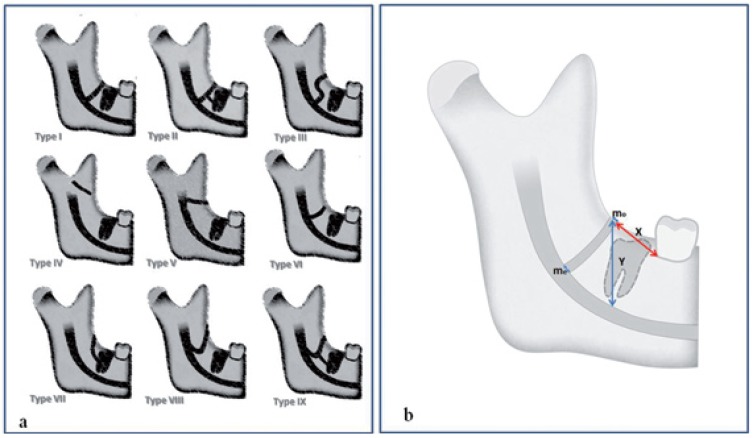
a) Schematic illustrations of different configurations of the retromolar canal. b) Schematic illustration of linear measurements taken of the retromolar canal; (X):horizontal distance from retromolar canal to second molar, (Y) height of retromolar canal, (C) width of retromolar canal, the width at the point of origin (wo) from the mandibular canal and the width at the point of exit (we) in the retromolar fossa in sagittal sections.

The retromolar canals were further classified into 9 categories according to the course and morphology. A schematic diagram of all types of retromolar canals is shown in figure 1.

Type I: Vertical course of retromolar canal [Narayana *et al*. 2002]; Type II: Vertical course of retromolar canal with additional horizontal branch [von Arx 2011],; Type III:Vertical course of retromolar canal and then coursing posterosuperiorly toward the retromolar fossa (by Present Study); Type IV: Temporal crest canal [by Ossenberg *et al*. 1987]; Type V: Curved course of retromolar canal branching mandibular foramen (by Present Study); Type VI:Curved course of retromolar canal branching mandibular canal (by Present Study).; Type VII: Retromolar canal running from the retromolar fossa and opening into the periodontal ligament space [Patil *et al*, 2013]; Type VIII: Running anteriorly for some distance and then coursing posterosuperiorly toward the retromolar fossa foramen (by Present Study).Type IX:Running anteriorly for some distance and then coursing postero-superiorly toward the retromolar fossa foramen with additional horizontal branch foramen (by Present Study).

The following linear measurements were taken by using CBCT images (Fig. 1): the horizontal distance from the midpoint of the retromolar foramen to the distal cement-enamel junction (CEJ) of the second molar, the vertical distance (height) from the midpoint of the retromolar foramen to the upper border of the mandibular canal, the width at the point of origin from the mandibular canal and the width at the point of exit in the retromolar fossa were measured in sagittal sections. The region of exit of retromolar canals in the retromolar fossa was noted in coronal sections by dividing the retromolar fossa into the buccal half and lingual half.

The diameters of the origin of the MRCs were measured in sagittal slices. The MRCs were assigned to four groups according to their diameters: 0-1 mm, 1-2 mm, 2-3 mm and ≥ 3 mm. The visibilities of the MRCs in relation to their diameter groups were also explored on the OPGs to determine whether an increase in their diameters affected their visibility.

The OPGs and CBCT images were examined by two dentomaxillofacial radiologists independently. All the measurements were carried out by the same person (AES). To study the precision of the linear measurements as described above taken with CBCT and measurements were repeated 2 weeks later, showing a high correlation. The first measurements were used for further analysis.

Statistical Analysis

Statistical analysis was performed using SPSS® v. 15 (SPSS Inc., Chicago, IL) software program. Pearson Chi square and t-tests was performed for statistical analysis among gender, localization and measurements. A *p* value of <0.05 was considered statistically significant.

## Results

OPGs and CBCT images of 632 subjects (341 females and 291 males, ranging from 15-58 years with a mean of 27.47 ± 8.74 years) were included in the study. Of these subjects examined with CBCT, 317 patients had undergone a unilateral examination (146; left, 171; right) and 315 patients had undergone a bilateral examination. Thus, a total of 947 hemimandibles were examined.

A total of 253 MRCs (144 left, 109 right)were detected with CBCT images (26.7%). Only 29 of these 253 MRCs were identified with the corresponding panoramic radiographs ( Table 1). More women (41.1%) than men (38.8%) and more left sides (27.7%, 144/519) than right sides (25.4%, 109/428) tended to have MRCs, but these differences did not reach statistical significance (*P* = .157 and *P* = .110, respectively) ( Table 1). Of the cases evaluated bilaterally, only 26 (8.25%) presented with a MRC on both sides. The interexaminer repeatability was tested and resulted in no significant statistical differences (*p*>.05).

**Table 1:**
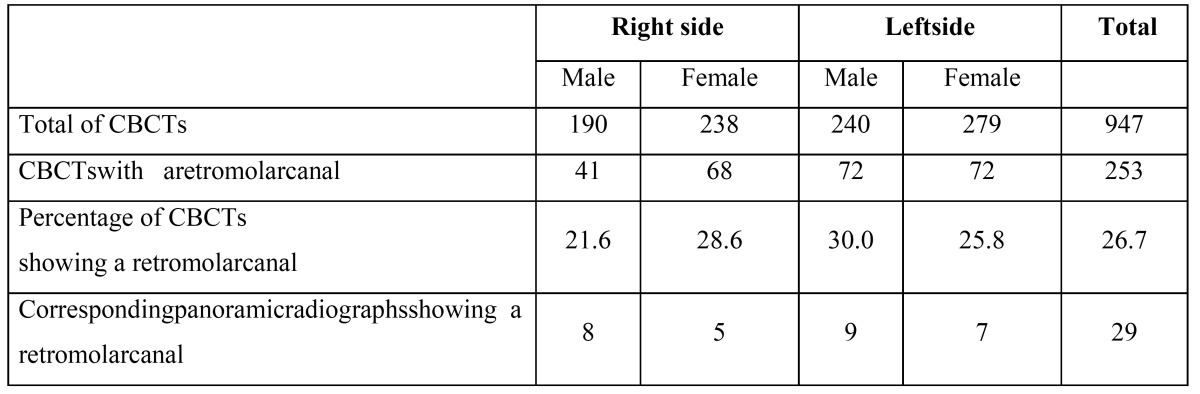
Radiographic Findingsper Sites (n =947).

**Figure 2:**
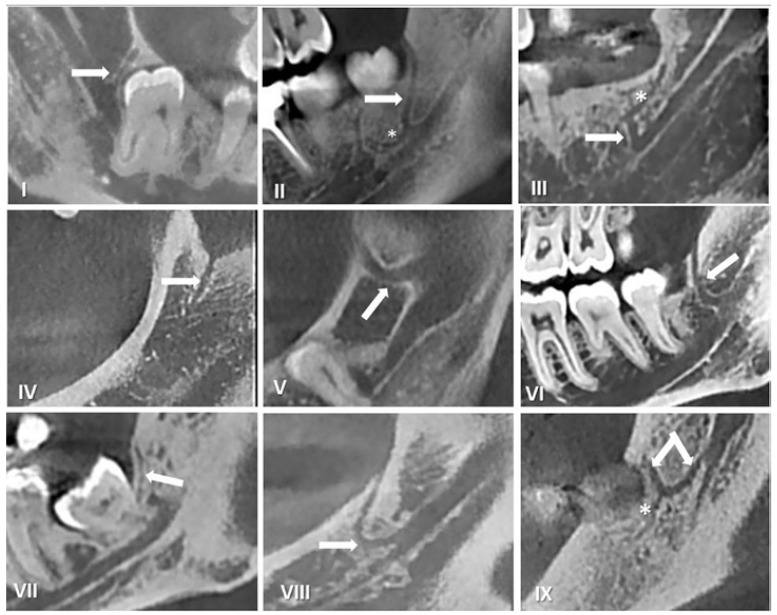
Different configurations of the retromolar canal observed on CBCT scan.

**Figure 3:**
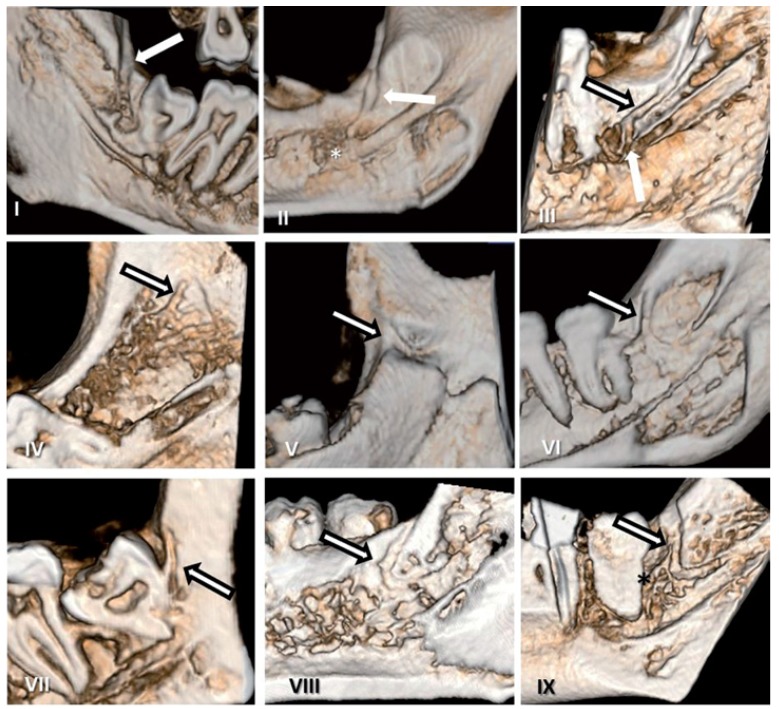
Corresponding sagittal CBCT cut of patients (which were shown in figure 2) indicates 3-D view of retromolar canal and its course.

With regard to canal morphology, most MRCs had a slightly curved (type VI, 28.46%), followed by vertical course (type I, 26.09%). (Fig. 2: I-IX and corresponding 3-D images were shown on fig. 3). Distribution of retromolar canals based on their types and region of exit in the mandible is also shown in  Table 2. The difference in the occurrence of retromolar canals between the right and left side in both unilateral and bilateral category was not significant.

**Table 2:**
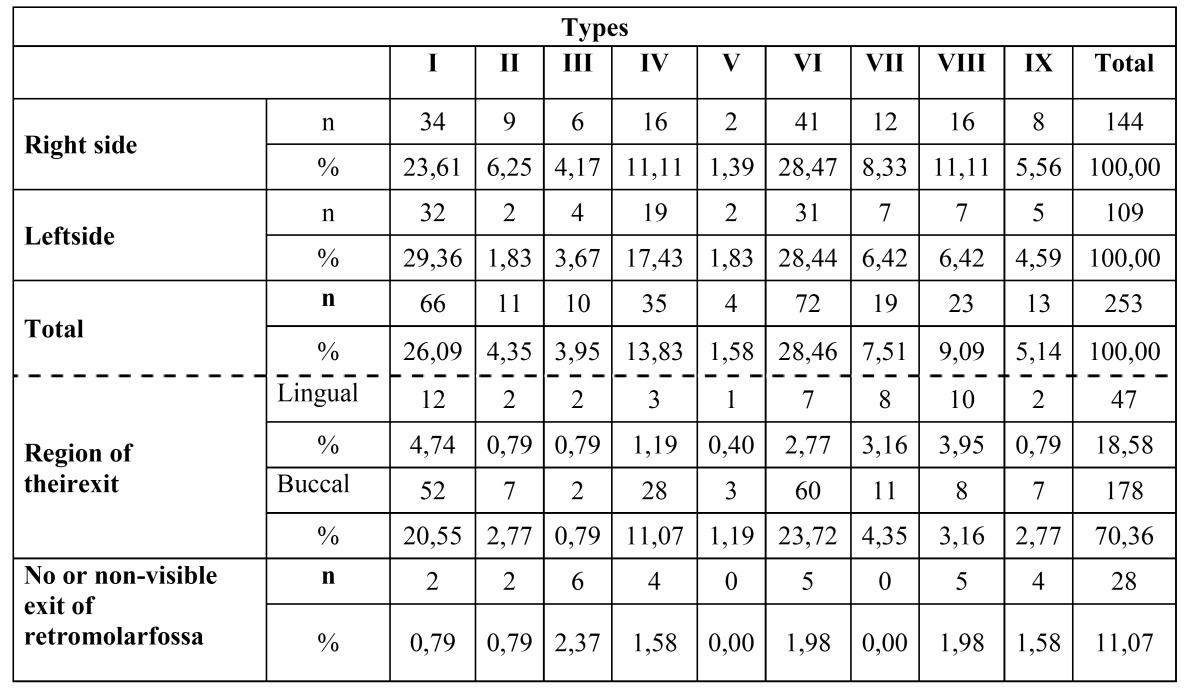
Distribution of retromolar canals with different types and region of exit in themandible (n = 253)

The distribution of subjects with different types of MRCs based on age groups and gender is shown in  Table 3. It was found that 113 of 430 sides and 140 of 517sides presented with retromolar canals in males and in females, respectively and hence, no difference in the occurrence of retromolar canals with regard to gender was evident.

**Table 3:**
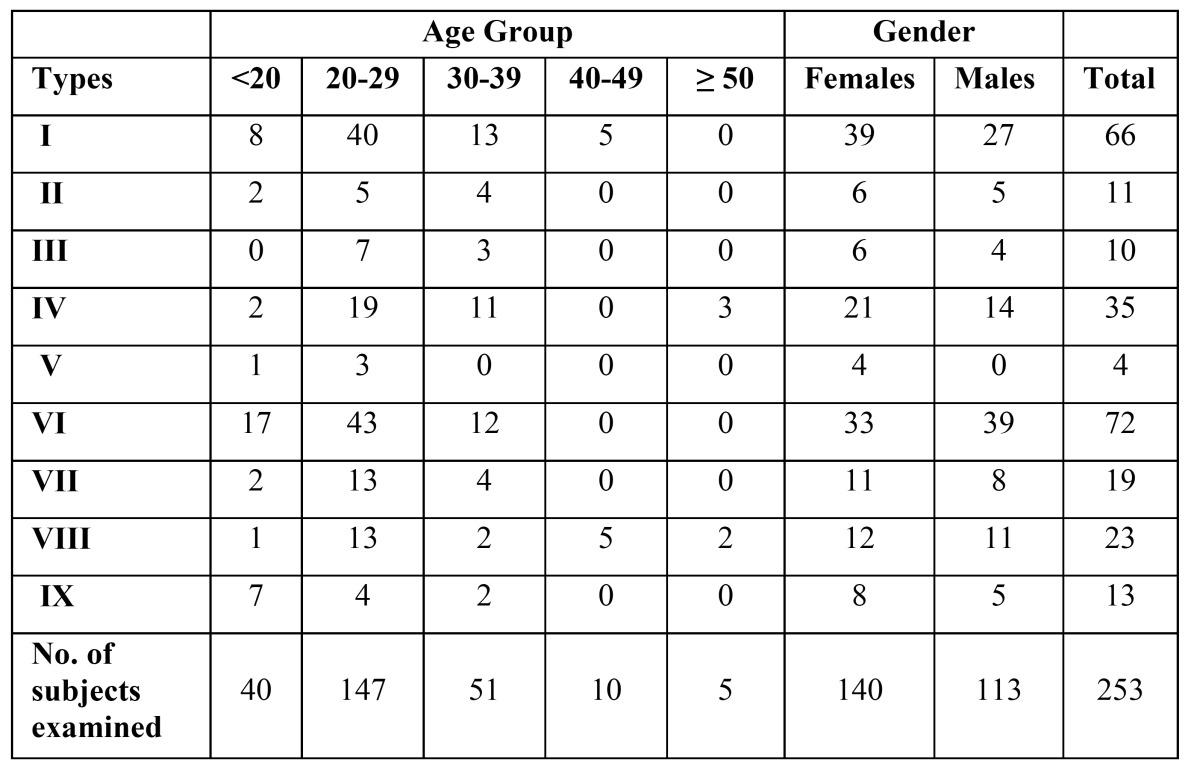
Distribution of subjects with different types of retromolar canals based on age and gender.

The linear measurements are summarized in  Table 4. The mean height of the canal (vertical distance from retromolar foramen to mandibular canal) was 11.4mm (± 2.61mm; range, 6.3-16.2 mm).The mean distance from the midpoint of the retromolar foramen to the distal aspect of the second molar was 15.45 mm (± 3.12 mm; range, 11.8-24.40 mm). Differences were found for these mentioned distance for gender (p= .014). The mean width at the point of origin from the mandibular canal and the width at the point of exit in the visible retromolar fossa (n=199) were 2.24 ± 0.94 mm and 1.64 ± 0.64 mm, respectively. Statistically difference were found for the width at the point of origin from the mandibular canal (*p*: .037), the mean distance from the retromolar canal to the second molar (*p*: .042) and height of MRC when compared the gender.

**Table 4:**
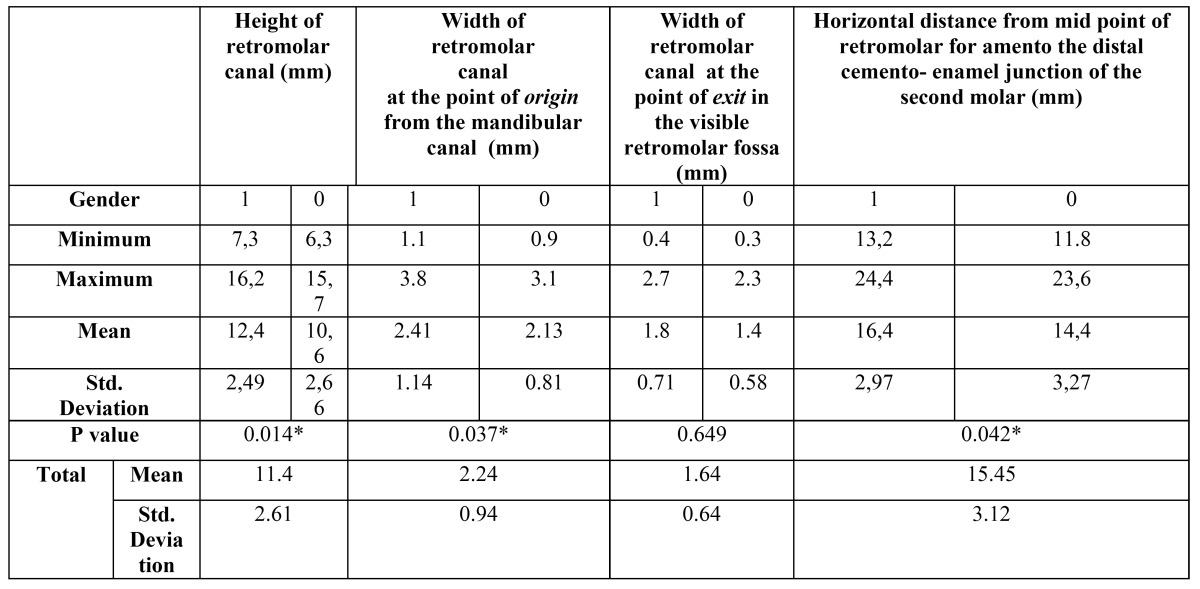
Height and weight of retromolar canal of 253 hemimandibles; distance from distal side of the alveolar socket of mandibular second molar tooth with their comparison between gender-side.

When considering the table of the diameter groups of the MRCs at the point of origin from the mandibular canal, the majority of them (n=216, 85.3 %) was between 1 and 3 mm (Group II and III) and the least of them (n=11, 4.35 %) was less than 1 mm (Group I). Besides, 26 (10.28%) MRCs had a diameter of greater than or equal to 3 mm (Group IV). Visibility of the MRC on OPGs, according to the diameter groups, was not statistically significant for both sides (*p*>.05).

## Discussion

The present radiographic study evaluated the presence and morphology of the retromolar canal in 632 patients by means of panoramic radiography and CBCT. The findings propose that the radiological frequency of the retromolar nerve is notable, with a possible relevance in all anesthetic and surgical approaches regarding the retromolar area and mandible.

Ossenberg stated that the MRC is an anatomic variant which normally arises from the mandibular canal behind the third molar and travels anterosuperiorly to the mandibular retromolar foramen (MRF), which is located in the retromolar fossa (10). Carter et al. demonstrated that a neural branch to the mandibular molars arises from the inferior alveolar nerve (IAN) or from the retromolar branch that travels through the MRC (11). However, Jablonski *et al*. (12) have shown an aberrant buccal nerve originating from the IAN within the ramous of the mandible, traversing through the MRC, emerging from the MRF, and then passing forward and upward to penetrate the buccinator muscle.

The importance of the retromolar foramen and canal with accessory innervation, but also with failure of locoregional anesthesia in dentistry (12). A cadaver study performed by Schejtman *et al*. (13) assessed the course of the neurovascular bundle originating from the MRF. After leaving the foramen, these elements were distributed mostly to the temporal tendon, the buccinator muscle, the most posterior zone of the alveolar process, and the third mandibular molar. Ikeda *et al*. (14) suggested that branches of the mandibular division of the IAN can arise high in the infratemporal fossa and extend to the base of the coronoid process to enter the mandible in the retromolar fossa and to allow sensory fibers to innervate the mandibular molars.

Previous studies on the prevalence of the MRC have been conducted by using cadaver mandibles, and those studies generally assessed the MRF rather than the MRC. The incidence of MRCs in osseous and CBCT studies has been found to range from 6.1%-72% among different populations (3,10,14-16). Ossenberg (10) reported that the occurrence rates of MRF differ between populations, with the rate in the North American population being relatively lower than those in other populations, such as those of Northeast Asia, Europe, and Africa. Naitoh *et al*. (2) investigated the MRC as a subtype of the bifid mandibular canal using clinical CBCT images (voxel size 0.155 mm) and observed the MRC at a frequency of 25.4% per mandible and 13.5% per side.

The contents of the MRC have been reported to consist of branches of inferior alveolar vessels and nerves (1,17). Histopathological surveys confirm the presence of myelinated nerve fibres associated with numerous venules and an artery (17). Schejtman *et al*. (13) performed the dissection of retromolar canal in cadavers during his autopsy, they found that the most constant element is a myelinated nerve (present in eight of the nine cases that were studied under a microscope).

As the present study has shown, the ability to detect a MRC with panoramic radiography is limited. Von Arx *et al*. (4) suggested thatone explanation might be that the MRC at 3 mm below the retromolar foramen is too thin to be detected (mean width, 0.99 mm; range, 0.5-1.75 mm). Kaufman *et al* (18) detected bilateral accessory canals, 1-2 mm in diameter, slightly anterior to the ascending ramus of the mandible. Bilecenoglu and Tuncer (3) reported mean distances of 4.2 mm and 11.9 mm from the MRF to the distal aspect of the alveolar socket of the third and second molars, respectively.

In conclusion, the present study demonstrated the presence of a retromolarcanal in 26.7% of CBCTs and in 3.06% of panoramic radiographs. The findings suggest that the MRC isn’t a rare anatomical structure. This study therefore clearly establishes the incidence and importance of the MRC. The detection of the presence of the MRC using CBCT may be crucial for extraction of mandibular third molar determined to be extremely close to the mandibular canal on panoramic radiographs. It is therefore important to confirm the course of the MRC and the location of the MRF prior to these surgical procedures.
